# Sex affects immunolabeling for histone 3 K27me3 in the trophectoderm of the bovine blastocyst but not labeling for histone 3 K18ac

**DOI:** 10.1371/journal.pone.0223570

**Published:** 2019-10-10

**Authors:** Luciano de R. Carvalheira, Paula Tríbulo, Álan M. Borges, Peter J. Hansen

**Affiliations:** 1 Department of Animal Sciences, D.H. Barron Reproductive and Perinatal Biology Research Program, and Genetics Institute, University of Florida, Gainesville, Florida, United States of America; 2 Departamento de Clínica e Cirurgia Veterinárias, Escola de Veterinária, Universidade Federal de Minas Gerais, Belo Horizonte, Minas Gerais, Brazil; School of Sciences and Languages, Sao Paulo State University (UNESP), BRAZIL

## Abstract

The mammalian embryo displays sexual dimorphism in the preimplantation period. Moreover, competence of the embryo to develop is dependent on the sire from which the embryo is derived and can be modified by embryokines produced by the endometrium such as colony stimulating factor 2 (CSF2). The preimplantation period is characterized by large changes in epigenetic modifications of DNA and histones. It is possible, therefore, that effects of sex, sire, and embryo regulatory molecules are mediated by changes in epigenetic modifications. Here it was tested whether global levels of two histone modifications in the trophectoderm of the bovine blastocyst were affected by sex, sire, and CSF2. It was found that amounts of immunolabeled H3K27me3 were greater (P = 0.030) for male embryos than female embryos. Additionally, labeling for H3K27me3 and H3K18ac depended upon the bull from which embryos were derived. Although CSF2 reduced the proportion of embryos developing to the blastocyst, there was no effect of CSF2 on labeling for H3K27me3 or H3K18ac. Results indicate that the blastocyst trophoctoderm can be modified epigenetically by embryo sex and paternal inheritance through alterations in histone epigenetic marks.

## Introduction

The mammalian embryo displays sex differences very early in development and long before gonadogenesis. There are dissimilarities between male and female embryos during the preimplantation period in gene expression [1–6], mitochondrial number [7], secretion of miRNAs [8], acute responses to specific embryokines [9], altered development in response to specific stresses [10,11], and long-term changes in the developmental program caused by changes in the microenvironment of the embryo [see 12,13 for review]. The key driver of differences between male and female embryos early in development, particularly before X-chromosome inactivation, is the unequal distribution of sex chromosomes. In the bovine, for example, about 50% of the genes differentially expressed between male and female embryos at the morula stage are located on the X chromosome [3] and 18–62% at the blastocyst stage [2,6]. It has been hypothesized that transcriptional and epigenetic changes driven by the sex chromosomes regulate autosomal chromosomes early in development to establish sex-specific patterns in the epigenome later in development [14]. Sex differences in degree of methylation at specific loci in the blastocyst have been identified in cattle [7].

The epigenome of the bovine embryo undergoes large-scale changes during the preimplantation period. Initially, global DNA methylation and the extent of various histone modifications (H3K27me3, H3K9ac, H3K18ac, and H3K4me3) decline in abundance to about the 8-cell stage before increasing thereafter to the blastocyst stage [15–18]. Other histone modifications, specifically H3K9me2, H4K5ac, and H4K8ac, do not decline during early cleavage stages but increase in abundance by the morula and blastocyst stage [18]. Here we tested the hypothesis that two modifications in histone H3 important for epigenetic regulation in the trophectoderm (TE) of the bovine blastocyst are modified by embryo sex. The modifications were trimethylation of lysine 27 (H3K27me3), which is associated with gene-specific silencing of transcription, and acetylation of lysine 18 (H3K18ac), which increases chromatin accessibility and transcriptional activity [19]. It was also tested whether CSF2, which can affect trophoblast function of male embryos differently than females [20], alters histone modifications in the TE in a sex-dependent manner. Additionally, it was hypothesized that sire would affect histone epigenetic marks in the trophectoderm of the blastocyst. This hypothesis is based on observations that the bull used to contribute spermatozoa for fertilization can have a large impact on competence of the resultant embryo to develop to the blastocyst stage [21] and can also affect DNA methylation in the blastocyst [22].

## Materials and methods

### Embryo production

Cumulus oocyte complexes (COC) were obtained by using a scalpel to slice open 2–8 mm diameter follicles on the surface of ovaries obtained at a local abattoir. Ovaries were obtained from cattle of a mix of undetermined genotypes. Most oocytes were from *Bos taurus* but some were collected from animals containing an unknown amount of *B*. *indicus* genetics. After scoring the surface of the ovary with the scalpel, the ovary was vigorously agitated in BoviPRO oocyte wash medium (MOFA Global, Verona, WI, USA) to release COC. Medium was then filtered with a 100 μm cell strainer (Corning, Corning, NY, USA) and the retained material was rinsed onto square petri dishes with oocyte wash medium. Using a dissecting microscope and a Wiretrol^®^ micropipette (Drummond, Broomall, PA, USA), COC with at least three layers of compact cumulus cells and homogeneous cytoplasm were selected and placed in groups of 10 in 50 μL drops of BO-IVM medium (IVF Bioscience, Falmouth, UK) under mineral oil. The COC were matured for 22–24 h at 38.5°C in a humidified atmosphere of 5% (v/v) CO_2_ in air.

Media for fertilization and embryo culture were prepared as described elsewhere [23] except that media were supplemented with 20 μg/mL amikacin (Sigma-Aldrich, St. Louis, MO, USA). Matured COC were washed in HEPES-TALP and transferred in groups of 30 into microdrops of medium consisting of 50 μL IVF-HEPES and 3.5 μL of a solution of 0.05 mM penicillamine, 0.25 mM hypotaurine, and 25 μM epinephrine covered by mineral oil. Each drop was fertilized with 20 μL X or Y-sorted semen that had been purified using Puresperm 40/80 as described by the manufacturer (Nidacon International, Mölndal, Sweden). The final concentration was ~ 2 x 10^6^ sperm/mL. After 12–18 h at 38.5°C in a humidified atmosphere of 5% (v/v) CO_2_ in air, presumptive zygotes were removed from fertilization drops and denuded by vortexing for 5 min in a tube consisting of 100 μL 10,000 U/mL hyaluronidase in 0.9% (w/v) NaCl and 600 μL of HEPES-TALP. The denuded putative zygotes were washed two times in HEPES-TALP, washed once in SOF-BE2, placed in groups of 25–30 in 45 μL drops of SOF-BE2 medium covered with mineral oil, and incubated at 38.5°C in 5% CO_2_, 5% O_2_, 90% N_2_ and humidified air in a benchtop incubator (EVE, WTA, College Station, TX, USA). At day 5 post-insemination [120 h after insemination (hpi)], 5 μL of SOF-BE2 containing either 100 ng/mL CSF2 (a gift from CIBA-GEIGY, Basle, Switzerland) or vehicle [Dulbecco’s phosphate-buffered saline (DPBS) containing 1 mg/mL fatty-acid free bovine serum albumin (BSA) (Sigma-Aldrich)] was added to each culture drop result in a final concentration of 10 ng/mL CSF2. Cleavage and blastocyst rate were evaluated at day 3.5 post insemination (84 hpi) and at day 7.5 (180 hpi), respectively. The blastocysts at 7.5 were harvested, fixed at in 4% (w/v) paraformaldehyde for 15 min and stored in 50 μL DPBS containing 1% (w/v) polyvinylpyrrolidone at 4°C until processing for histone labeling.

Embryos were produced in a total of 20 replicates involving 5907 oocytes. For each replicate, X and Y-sorted sperm from one or more bulls (ST Genetics, Navasota, TX, USA) were used to fertilize oocytes, and embryos were treated with CSF2 or vehicle. A total of three different Holstein bulls (ST Genetics, Navasota, TX, USA) was used in the experiment. Bull A was used in 8 replicates, bull B in 8 replicates and bull C in 7 replicates. For histone labeling, a total of 17 replicates (4789 oocytes) was performed and each replicate utilized sperm from a single bull for fertilization. Bull A was used in 6 replicates, bull B in 6 replicates and bull C in 5 replicates.

### Immunolabeling

Fixed blastocysts were permeabilized for 30 min at room temperature using 0.25% (v/v) Triton X-100 (Fisher Scientific, Waltham, MA, USA) diluted in DPBS. Embryos were washed three time in wash buffer [DPBS containing 1 mg/mL fraction V BSA (Sigma-Aldrich) and 0.1% (v/v) Tween 20] and then incubated for 1 h at room temperature in a blocking buffer consisting of DPBS containing 50 mg/mL BSA. Blastocysts were then incubated overnight at 4°C with one of three primary rabbit polyclonal antibodies at a concentration of 1 μg/mL: rabbit polyclonal anti-human histone H3 [ab18521; Abcam (Cambridge, MA)], anti-H3K27me3 [07–449, MilliporeSigma (Burlington MA)] and anti-H3K18ac (ab1191; Abcam). Antibodies were diluted with a buffer consisting of DPBS containing 0.1% (v/v) Tween 20 and 10 mg/mL fraction V BSA. As a negative control, primary antibody was replaced by 1 μg/mL rabbit IgG (PRABP01, Bio-Rad [Hercules, CA)]. Blastocysts were washed three times with wash buffer and incubated with 2 μg/mL 1:1000 (v/v) goat anti-rabbit IgG conjugated with Alexa 488 [A-11008, Invitrogen/ThermoFisher Scientific (Waltham, MA, USA)] for 1 h at room temperature. After washing three times in wash buffer, blastocysts were incubated with 1 μg/mL Hoescht 33342 (Sigma-Aldrich) for 15 min at room temperature to label nuclei, rinsed in DBPS containing 0.2% (w/v) polyvinylpyrrolidone, placed on a slide with 5 μL ProLong Gold Antifade Mountant (Invitrogen) and covered with a coverslip. Imaging was performed with an Axioplan 2 epifluorescense microscope (Zeiss, Göttingen, Germany) with 40x objective and green and blue filters. Digital images were acquired using AxioVision software (Zeiss) and a high-resolution black and white Zeiss AxioCam MRm digital camera. For each primary antibody, the exposure times were constant for all blastocysts analyzed in the same replicate.

### Image analysis

Image J software (version 1.51j8, National Institutes of Health, Bethesda, MD, USA) was utilized to quantify immunofluorescence. Blastocyst nuclei were identified and counted based on Hoechst 33342 labeling. The region of the inner cell mass (ICM) was identified as an aggregation of cells in the blastocyst and the remaining cells were considered as TE cells. Because of difficulties in distinguishing between ICM cells and overlying TE cells in the area of the ICM, analysis was performed only for nuclei that were clearly in the TE region. With the freehand selection tool, a perimeter was manually drawn around each individual nucleus to measure immunofluorescent intensity for H3, H3K27me3, or H3K18ac (green) as well as DNA (blue). Background intensity in the green channel was measured in a random dark area close to the embryo and this value was subtracted from the value for histone intensity. Mean intensity of nuclear labeling for H3, H3K27me3 and H3H18ac was determined for each blastocyst. The total number of blastocysts analyzed was 123 for H3 (17 replicates), 125 for H3K27me3 (17 replicates) and 128 for H3H18ac (16 replicates).

### Statistical analysis

Statistical analysis was performed using SAS software (version 9.4: SAS Institute Inc., Cary, NC, USA). Data on percent of oocytes that cleaved, percent of presumptive zygotes that developed to the blastocyst stage and percent of cleaved embryos that developed to the blastocyst stage were analyzed by the Glimmix procedure of SAS. The individual embryo was considered as the experimental unit. Responses were considered as binary variables and analysis was performed by logistic regression with a binary data distribution. The model included sex (male vs female), treatment (vehicle vs CSF2), bull (A, B, and C), interactions between these variables and replicate. The latter term was considered as a random class variable.

Data on blastocyst cell number and fluorescent intensity for H3 were analyzed by analysis of variance using the Mixed procedure of SAS. For cell number, blastocyst was considered the experimental unit and the model included sex, treatment, bull, and interactions as fixed effects and with replicate nested within bull as a random effect. The analysis for H3 was similar except that the fluorescent intensity of Hoechst labeling was used as a covariate to adjust for differences in nuclear condensation. Data on fluorescent intensity for H3K27me3 and H3K18ac were also analyzed using the Mixed procedure of SAS. The model included sex, treatment, bull, and interactions as fixed effects, replicate nested within bull as a random effect and with the average fluorescent intensity of H3 for that embryo’s bull-sex-treatment subgroup as a covariate to adjust for differences between groups in total H3 immunoflourescence. While results are not shown, conclusions were similar if H3 intensity was not used as a covariate.

## Results

### Embryonic development

**Fig 1:**
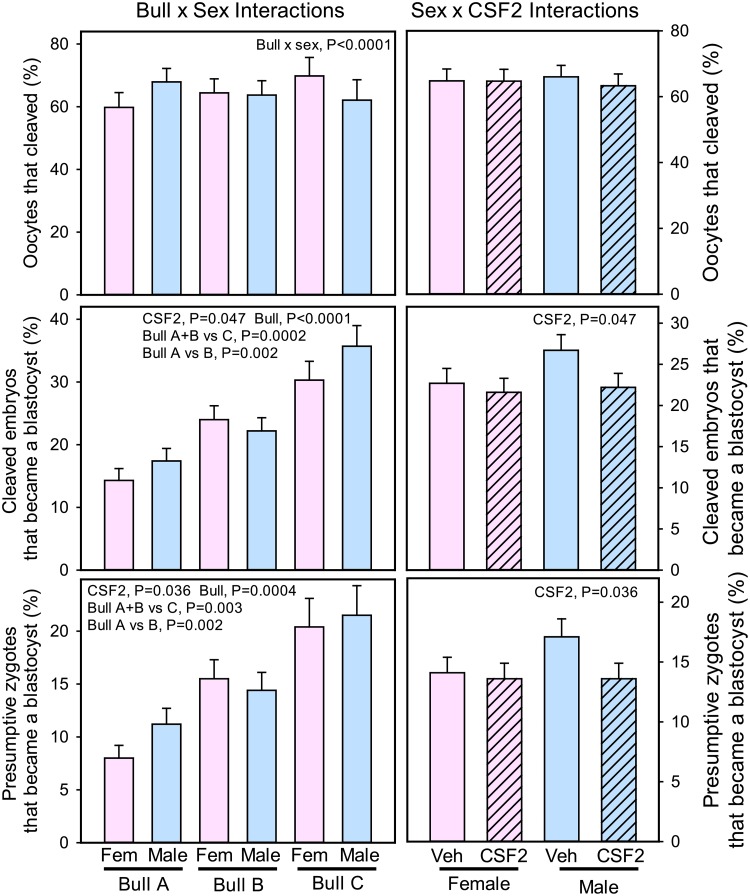
Competence of oocytes to cleave after fertilization and of embryos to develop to the blastocyst stage as affected by sex, bull and CSF2.

There was no effect of sex, CSF2, or bull on the proportion of oocytes that cleaved (Fig 1). There was, however, a bull x sex interaction (P<0.0001) that resulted because cleavage was greater for Y-sorted sperm for Bull A and greater for X-sorted sperm for Bull C and with no difference between semen types for Bull B.

There was no effect of sex on development to the blastocyst stage (Fig 1). Treatment with CSF2 reduced development to the blastocyst stage, whether expressed as percent of cleaved embryos (P = 0.047) or percent of putative zygotes (P = 0.036) (Fig 1). There was no significant interaction between CSF2 and sex but the effect of CSF2 tended to be more pronounced in male embryos. Bull significantly affected development to the blastocyst stage with development being greatest for Bull C, intermediate for Bull B, and least for Bull A.

There was a tendency (P = 0.060) for total cell number to be greater for male embryos (132.3 ± 4.6 cells) than female embryos (125.4 ± 5.0 cells) but there was no effect of CSF2 (129.0 ± 4.9 cells for vehicle vs 128.8 ± 4.8 cells for CSF2), bull (P = 0.549; 126.5 ± 8.5, 135.2 ± 6.0 and 124.9 ± 8.5 cells for bulls A, B and C, respectively), or interactions.

### Immunolabeling for histone H3

**Fig 2:**
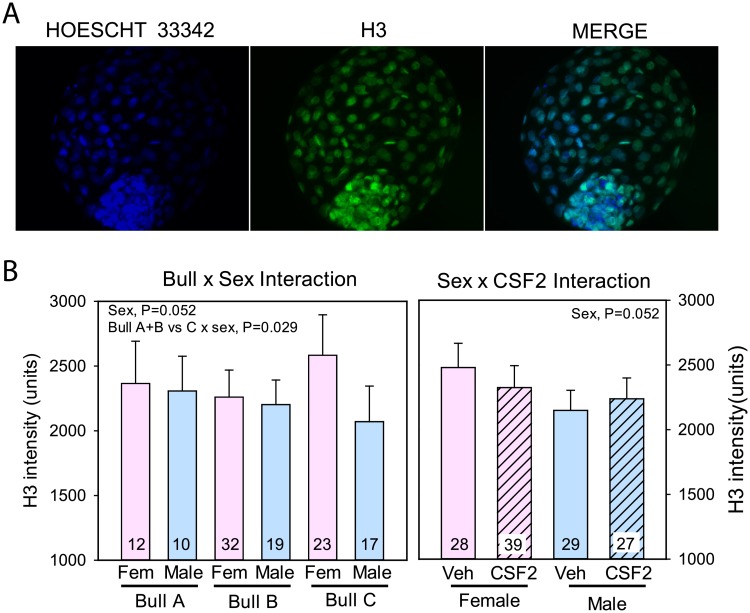
Immunolabeling for histone H3. A. Representative example of labeling of a blastocyst using antibody to H3. B. Least-squares means ± SEM of fluorescent intensity of H3 in nuclei of TE cells as affected by sex, bull, and CSF2.

Examples of immunolabeling for histone H3 are shown in Fig 2A whereas results for quantitative analysis of immunofluorescent intensity of labeling are shown in Fig 2B. As expected, labeling was limited to nuclei. There was no effect of CSF2, bull, or interactions between sex and CSF2 or bull and CSF2 on immunolabeling of histone H3. There was, however, an effect of sex (P = 0.052) and an interaction between bull and sex as determined by use of orthogonal contrasts. In particular, there was no effect of sex for Bulls A and B whereas labeling was greater for female embryos than male embryos for Bull C (Bulls A + B vs Bull C x sex; P = 0.029). Given this interaction, and to correct for possible differences in total histone H3 labeling between groups, subsequent analyses of immunolabeling for histone H3K27me3 and histone H3K18ac were performed using the average fluorescent intensity of histone H3 for an embryo’s bull-sex-treatment subgroup as a covariate.

### Immunolabeling for histone H3K27me3

**Fig 3:**
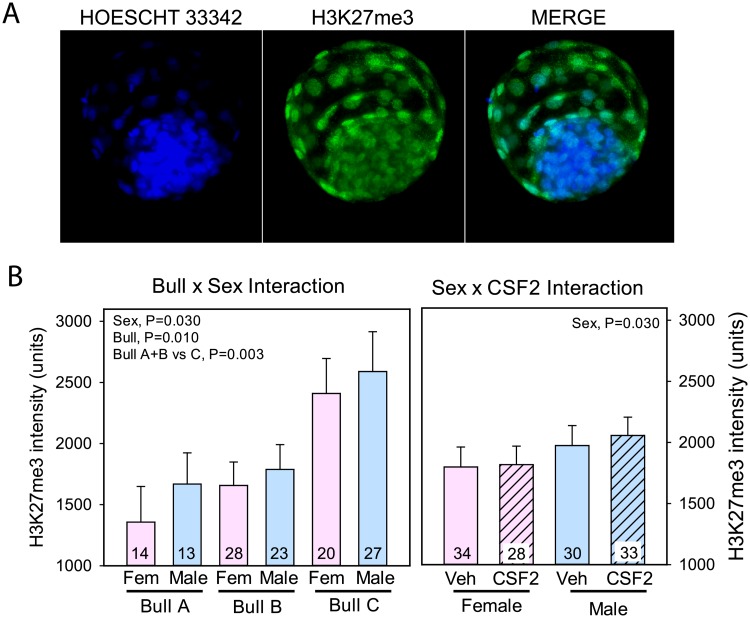
Immunolabeling for H3K27me3. A. Representative example of labeling of a blastocyst using antibody to H3K27me3. B. Least-squares means ± SEM of fluorescent intensity of H3K27me3 in nuclei of TE cells as affected by sex, bull, and CSF2 and after using labeling for H3 as a covariate.

Immunolabeling was limited to cell nuclei (Fig 3A). As shown in Fig 3B, amounts of immunolabeled H3K27me3 was greater for male embryos than female embryos (P = 0.030). Labeling was also affected (P = 0.010) by bull, with labeling greater for Bull C than Bulls A and B (P = 0.003). Labeling was not affected by CSF2 or any interaction with CSF2.

### Immunolabeling for histone H3K18ac

**Fig 4:**
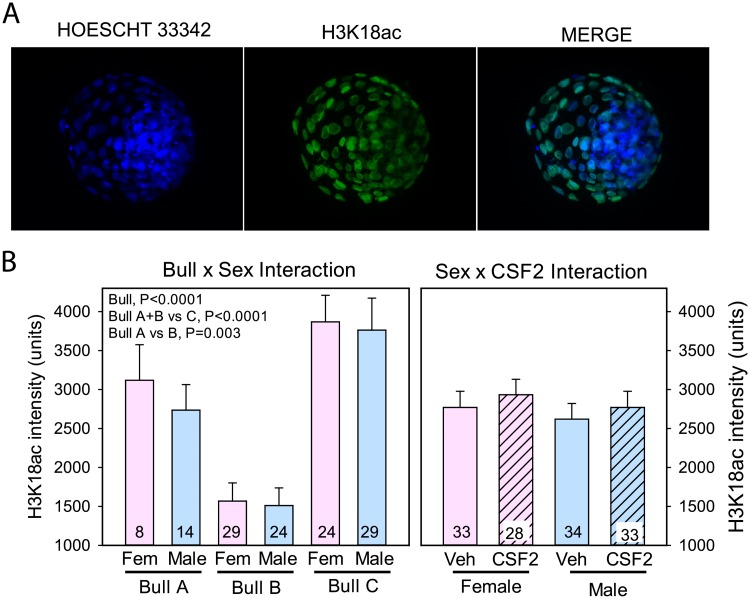
Immunolabeling for histone H3K18ac. A. Representative example of labeling of a blastocyst using antibody to H3K18ac. B. Least-squares means ± SEM of fluorescent intensity of H3K18ac in nuclei of TE cells as affected by sex, bull, and CSF2 and after using labeling for H3 as a covariate.

As for other histone marks, immunolabeling was limited to cell nuclei (Fig 4A). There was no effect of sex, CSF2, or the interactions with these factors on the amount of immunoreactive histone H3K18ac (Fig 4B). There was an effect of bull (P<0.0001), with labeling being highest for Bull C and least for Bull B (Bull A+B vs Bull C, P<0.0001; Bull A vs B, P = 0.003).

## Discussion

It is becoming well established that sex can affect developmental processes early in the life of the embryo, during the period leading up to development of the blastocyst. In the cow, sex differences during this period are observed in gene expression [2,3,6], mitochondrial DNA [7] and secretion of miRNAs [8]. Moreover, male embryos can respond differently to environmental conditions than female embryos as observed for effects of high concentrations of glucose on development to the blastocyst stage [11] and effects of serum and CSF2 on gene expression of cultured blastocysts [6, 9]. Present results are consistent with the idea that differences between sexes in characteristics of preimplantation development are due in part to differential epigenetic regulation of gene expression. This conclusion is based on the observation that the global amount of trimethylation of lysine 27 of histone H3 was greater for TE cells of male blastocysts than for female blastocysts. An additional finding was that the bull used to produce blastocysts also affected intensity of labeling for H3K27me3 and H3K18ac. These results indicate the importance of contributions of the sire to the epigenetic landscape of the developing embryo.

Differences between male and female embryos in establishment of H3K27me3 marks at the blastocyst stage could have important consequences for regulation of gene expression by developmental and environmental signals. In the cow, global levels of H3K27me3 are high in the oocyte and then decline to a nadir at the 8-cell stage through actions of Jumonji domain containing protein 3 before increasing thereafter to the blastocyst stage [18,24]. Knockdown of Jumonji domain containing protein 3 disrupts development to the blastocyst stage [24]. H3K27me3 domains in the chromatin of the preimplantation mouse embryo are not closely correlated with DNA methylation [25], suggesting an independent mechanism of transcriptional repression. The difference in degree of H3K27me3 marks between males and female embryos is not the result of X-chromosome inactivation in the female because this event has not yet occurred in the bovine blastocyst [2]. It should also be noted that differences between male and female embryos might be slightly greater than indicated from the current study because of the fact that about 10% of sexed semen contains sperm of the wrong sex chromosome [26].

In contrast to sex differences in degree of trimethylation of H3k27, there was no effect of sex on global amounts of H3K18ac or, in another study [27], H3K29ac. The lack of sex differences does not necessarily mean that these epigenetic marks are not involved in differences between male and female embryos during preimplantation development because of the possibility for locus-specific regulation. For buffalo embryos produced by somatic cell nuclear transfer, global levels of H3K18ac was greater for embryos derived from male somatic cells than female somatic cells [28].

Epigenetic differences between male and female embryos are likely to extend to DNA methylation. Using anti-methylcytosine to measure global DNA methylation, Dobbs et al. [15] observed that female embryos experienced greater DNA methylation at the 6–8 cell stage than male embryos. At the blastocyst stage, differences between sexes were reduced and DNA methylation was higher for male embryos [15]. Level of DNA methylation at one minisatellite region was greater for male blastocysts [7] and expression of DNA methyltransferases 3A and 3B were greater for males also [7].

As found earlier [21,29], there were differences between bulls in the competence of cleaved embryos to develop to the blastocyst stage. The bull contribution to the embryo includes both genetic and epigenetic information. Single nucleotide polymorphisms in specific genes have been associated with embryonic development [21,30,31]. There are also differences between bulls in the degree of DNA methylation and histone epigenetic modifications in sperm [22,32]. Differences in DNA methylation exist even among monozygotic twins [33]. However, it is unlikely that the differences in H3K27me3 and H3H18ac between embryos sired by different bulls reflects differences in degree of epigenetic modifications in sperm histones. This is so because paternal chromosomes become associated with maternally-derived histones coincident with syngamy [34] and there is an absence of immunoreactive H3K27me3 marks in the paternal pronucleus of the cow [18]. It is more likely that other transcriptional or epigenetic regulators that differ between bulls result in varying degree of histone H3 epigenetic marks.

There was no effect of CSF2 on patterns of labeling for H3K27me3 or H3K18ac in the blastocyst. This is despite the fact that CSF2 can act on the bovine embryo from day 5 to 7 of development to program trophoblast elongation at day 15 of gestation [20] and gene expression in placenta and liver at day 86 of gestation [35]. Effects of CSF2 on elongation depend on sex because CSF2 increased trophoblast length in males but decreased it in females [20]. Some long-term consequences of effects of CSF2 on the embryo likely involve changes in gene expression [9,36]. In addition, CSF2 can induce epigenetic changes in the embryo as evidenced by sex-dependent changes in the degree of DNA methylation at hundreds of loci in the trophoblast of the day 15 embryo [20]. The failure to find large-scale changes in histone epigenetic modifications does not preclude the possibility that CSF2 regulates histone modifications because only two histone epigenetic marks were evaluated and because the analytic procedure used does not allow examination of histone modification at specific genomic regions.

Treatment with CSF2 decreased the percent of embryos becoming blastocysts. The effect of CSF2 on competence of embryos to develop to the blastocyst stage is highly variable and dependent on the overall level of competence of embryos for development. When the percent of control embryos becoming blastocysts was low, CSF2 increased blastocyst percent whereas CSF2 decreased blastocyst yield when the percent of embryos becoming blastocysts in the control group was high [37]. It is possible that CSF2 promotes development to the blastocyst stage by reducing consequences of cellular stress. Indeed, CSF2 can reduce the degree of apoptosis in bovine embryos caused by heat shock [38]. The reason why CSF2 is inhibitory to development when embryonic competence is high is not known but perhaps activation of mechanisms that promote development in a stressed embryo are deleterious when cellular conditions for development are more favorable.

In conclusion, the degree of trimethylation of lysine 27 of histone H3 in the TE of the bovine embryo depends upon embryo sex. Moreover, sire can affect HeK27me3 and K3K18ac. These results indicate that function of the blastocyst can be modified epigenetically by embryo sex and paternal inheritance in a manner dependent upon alteration of histone epigenetic marks. Identification of specific genes regulated by H3K27me3 and H3K18ac in the bovine embryo and consequences of variation in H3K27me3 and H3K18ac between embryos for developmental phenotype are important topics of future research. In addition, the study should be expanded to identify sex effects on other histone epigenetic modifications in both ICM and TE.

## Abbreviations

BSA: bovine serum albumin
COC: cumulus-oocyte complex
DPBS: Dulbecco’s phosphate-buffered saline
HEPES-TALP: HEPES -Tyrode’s albumin lactate pyruvate
ICM: inner cell mass
IVF-TALP: in-vitro fertilization-Tyrode’s albumin lactate pyruvate
SOF-BE2: synthetic oviduct fluid—bovine embryo 2
TE: trophectoderm
